# The sweet smell of chemistry: from terpene genes to strawberry aroma

**DOI:** 10.1093/plphys/kiag345

**Published:** 2026-06-04

**Authors:** Praveen Khatri, Marcella Teixeira

**Affiliations:** Assistant Features Editor, Plant Physiology, American Society of Plant Biologists; Department of Biology, University of Toronto at Mississauga, Mississauga, ON L5L 1C6, Canada; Assistant Features Editor, Plant Physiology, American Society of Plant Biologists; Hermiston Agricultural Research and Extension Center, Oregon State University, Hermiston, OR 97838, United States

The cultivated strawberry (*Fragaria* × *ananassa*) is an allo-octoploid fruit crop that arose from hybridization between the North American species *Fragaria virginiana* and the South American species *Fragaria chiloensis*, following earlier polyploidization events involving several diploid progenitors, including *Fragaria vesca* (Edger et al. 2019). This complex evolutionary history has shaped the genetic diversity of modern strawberry and provides an important backdrop for understanding its chemical diversity.

Strawberry is a widely appreciated fruit crop worldwide, and its popularity is rooted not merely in its sweetness and texture, but also in its highly complex aroma bouquet. One might wonder how this small fruit can offer such rich sensory information. Part of the answer lies in the more than 300 volatile organic compounds (VOCs) characterized in strawberry fruit, among which terpenes are major contributors to aroma complexity (Pyysalo et al. 1979; Bood and Zabetakis 2002). In cultivated strawberry, terpenes contribute to fruity, floral, and jasmine-like notes, whereas in wild relatives they contribute to more pungent floral, woody, or minty aroma blends (Aharoni et al. 2004; Urrutia et al. 2017). However, despite terpene aroma being the subject of numerous breeding programs, the biosynthetic basis of such diversity has remained only partially understood.

Terpene synthases (TPSs) are well known as major drivers of terpene diversity in plants. Terpenes occur in related families that are often grouped by carbon number, including C10 monoterpenes, C15 sesquiterpenes, and C20 diterpenes (Fig. 1). TPS enzymes generate this diversity by converting a small set of common precursors into a much broader range of terpene structures (Chen et al. 2011; Karunanithi and Zerbe 2019). In principle, the octoploid history of cultivated strawberry should provide fertile ground for TPS diversification.

**Figure 1:**
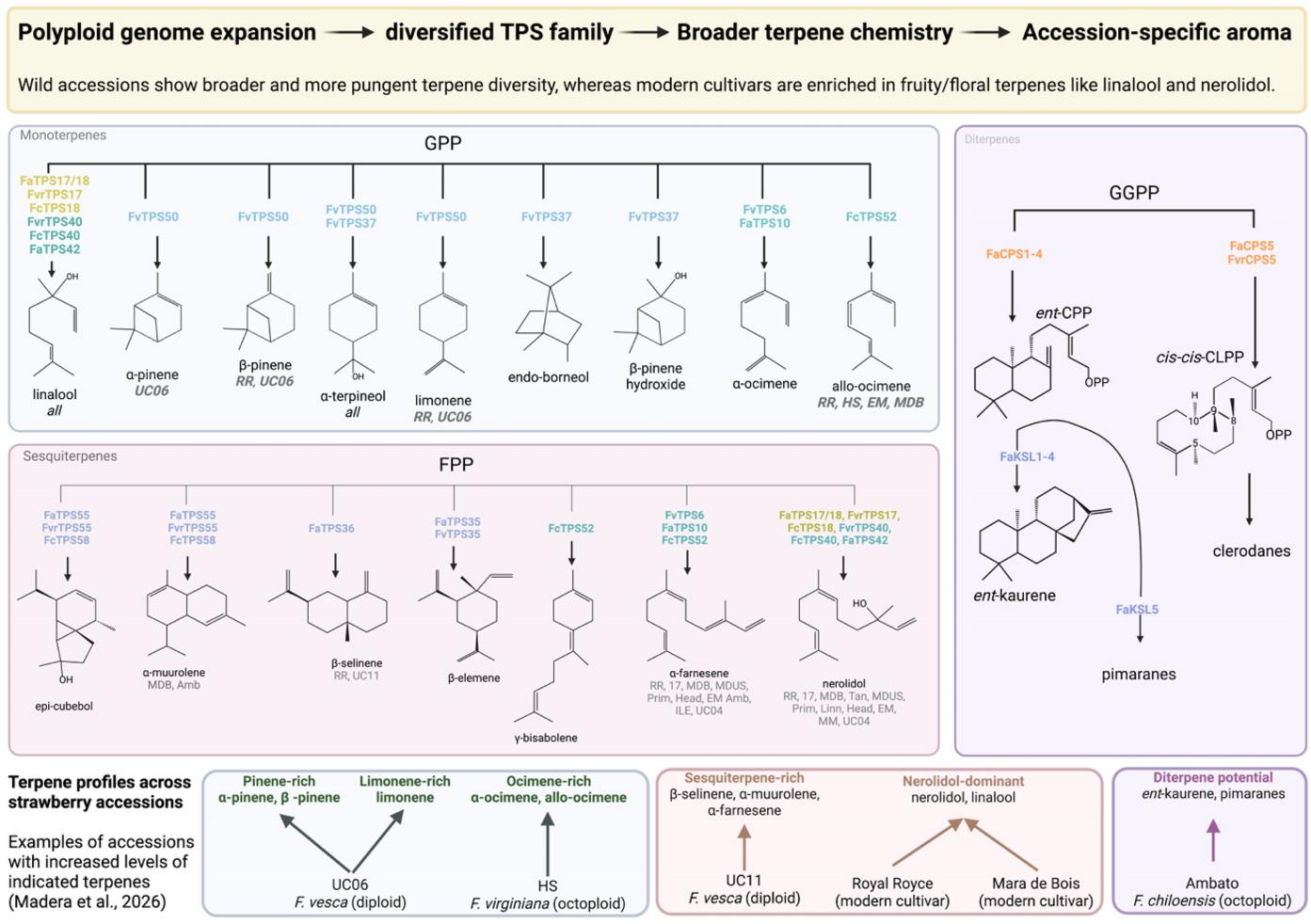
A model of the strawberry terpene-metabolic network. A simplified biosynthetic model of the major terpene products produced by the 27 functionally characterized strawberry terpene synthases (TPSs). In the top panels, the major branches producing monoterpenes, sesquiterpenes, and diterpenes from GPP, FPP, and GGPP and representative TPSs of each class are shown (GGPP, geranylgeranyl diphosphate; GPP, geranyl diphosphate; FPP, farnesyl diphosphate; CPP, copalyl diphosphate; CPS, copalyl diphosphate synthase; KSL, kaurene synthase-like). Enzymes are color-coded by TPS subfamily. The lower panels present characteristic terpene profiles of selected strawberry accessions. The model highlights a broader biological framework of how polyploid genome expansion contributed to TPS family diversification, enabling broader terpene chemistry and ultimately accession-specific aroma traits. Wild accessions generally show broader and more pungent terpene diversity, whereas modern cultivars are enriched in fruity and floral terpenes such as linalool and nerolidol. Where mentioned, accession abbreviations identify fruits where the respective terpenes were identified (RR, “Royal Royce”; 17, “17C224P011”; MDB, “Mara des Bois”; MDUS, “MDUS 5130”; Tan, “Tangi”; Prim, “Primella”; Head, “Headliner”; EM, “EarliMiss”; Amb, “Ambato”; ILE, “Isle de Lemuy 02A White”; HS, “Harris Springs”; MM, “Madame Moutot”). Adapted from Madera et al. (2026), Fig. 9. Created in BioRender. Teixeira, M. (2026) https://BioRender.com/g81x7b2.

TPSs convert conserved prenyl diphosphate precursors into a much larger repertoire of mono-, sesqui-, and diterpene scaffolds through carbocation-mediated cyclization and rearrangement reactions. Indeed, previous work identified a few key strawberry aroma synthases, including the dual linalool/nerolidol synthases FaNES1 and FaNES2 in cultivated strawberry, *F.* × *ananassa* (Fa), and the pinene synthase FvPINS in diploid *F. vesca* (Fv) (Aharoni et al. 2004; Fan et al. 2022). The impressive terpene chemical diversity found across modern cultivars, heirlooms, and wild accessions suggests the existence of additional, uncharacterized enzymes.

In this issue of *Plant Physiology*, Madera et al. (2026) address this gap by combining a genome-wide perspective of the TPS family involved in strawberry terpene metabolism. Using transcriptomics, metabolomics, and biochemical characterization across diverse strawberry accessions, the authors demonstrate how polyploid genome evolution, diversification of enzyme functions, accession-specific metabolite phenotypes, and fruit ripening jointly contribute to the formation of the terpene chemistry of strawberry aroma.

First, the genome mining of *F.* × *ananassa* “Royal Royce” revealed 104 TPS-like candidates, of which 75 were full-length TPS genes, with additional transcriptome analysis of cultivated and wild accessions further expanding this functional landscape. These genes were widely dispersed throughout the octoploid genome, with major concentrations on chromosomes 3 and 6. Synteny analysis indicated that some TPSs are retained as sets of homeologs, whereas others are apparently the result of tandem duplication or lineage-specific expansion. The resulting family contains representatives of the TPS-a, TPS-b, TPS-g, TPS-c, and TPS-e/f subfamilies and brings together mono-, sesqui-, and diterpene synthases within a single genomic framework. This expanded TPS gene family shows that terpene metabolism in strawberry is not constructed around a few exceptional enzymes, but rather around a large, diversified TPS family whose architecture reflects both polyploid history and metabolic specialization.

The strawberry TPS family is far more functionally diverse than previously appreciated. Biochemical characterization of 33 selected TPSs showed that 31 enzymes had measurable catalytic activity, underscoring the functional breadth of the strawberry TPS family. Besides confirming previously described nerolidol/linalool synthases found in modern cultivars, this biochemical characterization revealed additional enzymes producing representative monoterpenes and sesquiterpenes, including pinene, limonene, ocimene, farnesene, and humulene (Fig. 1). Madera et al. (2026) further reported the detection and biosynthesis of 47 terpene metabolites, including several that had not been previously described in strawberry, thereby expanding the known repertoire of terpene diversity (Fig. 1). These results show that TPS family in strawberry is not a narrow fruit-aroma module. It is a metabolically flexible, evolutionarily expanded system.

Further, to explore how TPS functional diversity translates into aroma variation, the authors performed metabolomics across a diversity panel of 96 strawberry accessions, cultivars, and wild ecotypes. This analysis showed that terpene aroma chemistry differs strongly between domesticated and wild germplasm. The metabolomics analysis identified 171 VOCs, including 31 terpene metabolites, and highlighted linalool, nerolidol, and α-terpineol as some of the most significant compounds distinguishing aroma profiles across the panel. The metabolite patterns further enhanced the distinction between domesticated and wild germplasm. The typical terpene profiles in modern *Fragaria* × *ananassa* cultivars were often dominated by linalool and nerolidol, while wild or diploid accessions often displayed more chemically diverse and sometimes more pungent terpene profiles. For example, the wild diploid accession *F. vesca* “UC06” stood out for monoterpene abundance, while the wild octoploid progenitor *F. virginiana* “UC11” was notable for sesquiterpene diversity. These metabolite maps are a nice illustration of the value of a genome-wide TPS survey: the field can now start to map accession-specific aroma chemistry to defined enzyme functions instead of treating volatile diversity as a black box (Fig. 1).

Importantly, this study avoids an overly simple gene-to-metabolite narrative. The authors found that the transcript abundances of TPS genes do not display a strong overall correlation with terpene profiles across accessions. Instead, terpene production during ripening reflects coordinated modulation of precursor pathways, *TPS* expression, cultivar-specific metabolic context, and developmental state that differ between cultivars. It also links gene family evolution with crop quality, showing that expansion of the strawberry TPS family helped generate both redundancy in major aroma compounds and accession-specific terpene diversity. The TPS family comprises not just a compendium of known enzymes; it is also an evolutionary substrate upon which breeding has acted, either intentionally or accidentally, to sculpt strawberry aroma. As such, this work provides a much-needed biochemical foundation for understanding how genomic variation translates into aroma-relevant chemistry.

The central advance of this study is the establishment of a biochemical framework that connects genome evolution, aroma variation, and breeding potential in strawberry. In the future, it will be exciting to understand how terpene production is affected by substrate availability, competition between branches, downstream oxidation and acylation, subcellular localization, and allele-specific regulation across different cultivars. Beyond advancing basic knowledge, this research provides a useful resource for breeding, moving from marker-trait association to the selection of lines with desirable biochemical capabilities. For a crop in which flavor has emerged as a central breeding goal (Fan et al. 2022), this represents a great leap forward. Strawberry aroma has long been appreciated for its complexity. This study now shows that its terpene complexity is not only rich, but predictable and engineerable.

## Recent related articles published in *Plant Physiology*

Wei et al. (2025) showed that the production of linalool in the detached peach fruit is controlled by a light-responsive module whereby the UV-B promotes PpCOP1-dependent degradation of PpMADS2, which controls the biosynthesis of linalool mediated by PpTPS1 and connects environmental signaling to fruit aroma metabolism.

Zheng et al. (2024) demonstrated that integrative multi-omics profiling could be effectively used to resolve the genetic basis of fruit aroma, as comparative analyses of yellow and purple passion fruit showed terpene variation to be linked to specific terpene synthase complements and functional characteristics of 12 TPSs associated with aroma differences between the 2 varieties.

## Data Availability

No data were generated in this study.
